# Integrins as Therapeutic Targets: Successes and Cancers

**DOI:** 10.3390/cancers9090110

**Published:** 2017-08-23

**Authors:** Sabine Raab-Westphal, John F. Marshall, Simon L. Goodman

**Affiliations:** 1 Translational In Vivo Pharmacology, Translational Innovation Platform Oncology, Merck KGaA, Frankfurter Str. 250, 64293 Darmstadt, Germany; sabine.raab@merckgroup.com; 2 Barts Cancer Institute, Queen Mary University of London, Charterhouse Square, London EC1M 6BQ, UK; j.f.Marshall@qmul.ac.uk; 3 Translational and Biomarkers Research, Translational Innovation Platform Oncology, Merck KGaA, 64293 Darmstadt, Germany

**Keywords:** integrin, therapy, clinical trial, efficacy, health care economics

## Abstract

Integrins are transmembrane receptors that are central to the biology of many human pathologies. Classically mediating cell-extracellular matrix and cell-cell interaction, and with an emerging role as local activators of TGFβ, they influence cancer, fibrosis, thrombosis and inflammation. Their ligand binding and some regulatory sites are extracellular and sensitive to pharmacological intervention, as proven by the clinical success of seven drugs targeting them. The six drugs on the market in 2016 generated revenues of some US$3.5 billion, mainly from inhibitors of α4-series integrins. In this review we examine the current developments in integrin therapeutics, especially in cancer, and comment on the health economic implications of these developments.

## 1. Introduction

Integrins are heterodimeric cell-surface adhesion molecules found on all nucleated cells. They integrate processes in the intracellular compartment with the extracellular environment. The 18 α- and 8 β-subunits form 24 different heterodimers each having functional and tissue specificity (reviewed in [1,2]). Most have multiple activation states, heterogeneous glycosylation and many have multiple splice variants, whose biological relevance is often unknown [2,3,4,5]. Integrins bind their ligands (extracellular matrix proteins or membrane-associated proteins on other cells) often through recognition of short amino-acid sequences—arginine-glycine-aspartic acid (RGD)—is a frequent recognition motif [6,7]. They are bi-directional signaling molecules. On one hand, they transduce information (“outside-in”) from the extracellular environment to modulate cell responses, including adhesion, spreading, migration, growth signaling, survival signaling, secretion of proteases and invasion [1,8,9]. On the other, their active state is modulated from the cytoplasm (“inside-out”) dependent on intracellular biochemistry [10,11]. Evidence is emerging that integrins specifically incorporated into both intracellular and extracellular vesicles are also involved in signaling [5,12].

Thus, since their discovery the possibility that integrins might promote pathogenic processes, including cancer, marked them as potential therapeutic targets. Meanwhile, their molecular structure, with crucial ligand binding and regulatory sites exposed to parenteral small and macromolecular drugs made them accessible drug candidates. As we shall see, these hopes have been realized with efficacious therapeutics targeting integrins. But also, regrettably, with very many less successful clinical trials.

In this review we provide an update on the progress of integrin-specific drugs in late-stage clinical trial. Two targeting concepts form the basis for integrin therapies: inhibition of integrin function; and the use of integrin expression patterns to specify drug delivery, either as addressed microencapsulated drug [13], or in the form of affinity-binder-drug conjugates [14]. Direct inhibition of integrin function has so far dominated therapeutic strategies that have reached the clinic, and is the only form of anti-integrin therapy so far shown to work in patients, so we focus here on this therapeutic form. However, first we will briefly review pre-clinical data that suggests potential novel clinical targets.

### 1.1. Issues in Translating Integrin Preclinical Data to the Clinic

Pre-clinical data frequently do not predict success in the clinic, as illustrated by the arduous pilgrimage of cancer therapies targeting the αvβ3 and αvβ5 integrins. Increased αvβ3 expression on clinical melanoma and glioblastoma is associated with invasive growth and poor survival [15,16,17]. Early preclinical studies reported that tumor-angiogenic blood vessels upregulated integrins αvβ3 and αvβ5, which promoted their migration and growth [18,19,20]. Since solid cancers generally develop their own vasculature to allow their progression beyond a small tumor (2 mm diameter) [21], suppressing angiogenesis seemed logical as a therapy for cancer [22]. Furthermore, inhibition of αvβ3 and αvβ5 suppressed xenografted tumor growth in chick egg and mouse models [18,19], so blocking αvβ3/5-function in humans seemed a viable therapeutic strategy. Anti-αvβ3 inhibitory antibodies, based on humanized form of the mouse monoclonal LM609 (etaracizumab; abegrin; vitaxin [23,24]) were tested clinically, and were well-tolerated. However, etaracizumab has now been tested in several trials for solid tumors but with little therapeutic effect (as discussed below).

Indeed, the role of αvβ3 at least has proved to be extremely complex: Reynolds and colleagues studying genetically modified mice (GEMMs) deficient for αvβ3 and/or αvβ5 discovered that neither were essential for tumor angiogenesis. In fact, lack of αvβ3 could *enhance* angiogenesis and tumor growth since, in the GEMM model, αvβ3 suppressed expression of VEGFR2, a receptor for a major endothelial cell growth factor, VEGF. Absence of αvβ3 allowed increased VEGFR2 expression, and stimulated blood-vessel growth [25,26]. Furthermore, sub-inhibitory doses of the αvβ3/β5-inhibitory peptide cilengitide promoted angiogenesis and tumor growth in animals [27]. Despite caveats that deletion of a receptor is not the same as its pharmacological blockade, and that simple animal xenograft models neither closely reflect human tumors genetically, nor with respect to pharmacokinetic and pharmacodynamic behavior, these data were disturbing. Indeed, in animal models, increased angiogenesis and vascular leakiness can enhance intratumoral delivery of conventional drugs, and improve treatment efficacy [28]. This apparently reverses the widely accepted clinical dogma, that inhibiting angiogenesis can enhance cancer therapy. The variable clinical efficacy with the efficacious anti-angiogenic drug bevacizumab highlights how context-specific anti-angiogenic cancer therapy may be [29,30]. Though VEGF can drive tumor angiogenesis in many preclinical models, in clinical practice other tumor angiogenic factors may be present, which make anti-VEGF therapy much less effective. In summary, despite initial promising pre-clinical data, targeting αvβ3 has so far failed in the clinic, likely in part due to insufficient knowledge of its biology. Nevertheless, these data may yet enable novel strategies based on αvβ3. Moreover, despite conflicting preclinical data, many studies still therapeutically target endothelial αvβ3 (reviewed in [31]).

In fact, so far few anti-integrin drugs designed to target epithelial or endothelial cells have significantly benefited patients, whereas several that target leukocytes or platelets have succeeded (examples are presented below, and reviewed in [2]). Whether this reflects a greater accessibility of blood-borne cells to intravenous therapies over cells of solid tissues remains to be determined. However, emerging data on integrins in the pathology of fibrosis and cancer suggests this balance may change. This is due to the unexpected and rapidly expanding picture we have about αv-integrins in the localized activation of TGFβ- family cytokines.

### 1.2. TGFβ Activation and Integrins: An Emerging Strategy?

TGFβs are pleiotropic cytokines that are locally activated during tissue remodeling to conduct a concert of repair processes including trans-differentiating fibroblasts into a contractile, collagen-producing myofibroblast phenotype; promoting angiogenesis; and suppressing immune response [32,33,34]. TGFβ1 also suppresses normal epithelial cell proliferation during repair, and is thus considered a tumor suppressor [32,33,34]. However, excess TGFβ activity can result in life-threatening tissue fibrosis, and it has thus long been a target for therapeutic intervention. Yet, some effective TGFβ signaling inhibitors, including ligand-traps and TGFβ receptor (TGFβR) kinase inhibitors are toxic, and can even enhance development of skin cancer (reviewed in [34,35]). Although drugs directly targeting the TGFβ signaling pathways are in development [36], novel TGFβ- therapeutic strategies dependent on integrins are emerging.

TGFβ is deposited by somatic and tumor cells in an inactive form, latent-TGFβ (LTGFβ). This is bound to the extracellular matrix (ECM) in a protein complex (reviewed in [37]). The initial LTGFβ protein complex is post-translationally processed so that TGFβ, bound to its protective pro-peptide, latency associated peptide (LAP), forms a homo-dimer. Several seminal papers have shown that αv integrins, and particularly αvβ6 and αvβ8, can mechanochemically help activate LTGFβ1 + 3, initially through high-affinity recognition of an RGD amino-acid motif in the LAPs of TGFβ1 and TGFβ3 [38,39]. Antibody-blockade of αvβ6 can suppress TGFβ-dependent bleomycin- [38] and radiation-induced lung fibrosis [40] and kidney fibrosis [41] in mouse models. This led Biogen-Idec to develop a humanized αvβ6-inhibitory antibody, STX-100, as a treatment for Idiopathic Pulmonary Fibrosis (IPF) (ClinicalTrials.gov: NCT01371305). Recently, both αvβ8-blocking antibodies [42], and αvβ1-blocking small molecules have been shown preclinically in mice to suppress fibro-inflammatory lung [43,44] and renal fibrosis [45], and the αvβ1-inhibitor also inhibits carbon tetrachloride-induced liver fibrosis [43]. These studies showed that by inhibiting local- rather than systemic -activation of LTGFβ it was possible to combine efficacy with low off-target toxicity.

Activated TGFβ is implicated in promoting late-stage cancer development and spread. Cancer cells often accumulate mutations or deletions in biochemical pathways that protect them from its growth inhibitory effects [32,33,34]. Indeed, high expression of αvβ6 in human carcinomas directly correlates with poor overall survival [46,47,48,49,50,51]. We recall that αvβ6 can locally activate LTGFβ1 + 3. Antibody-blockade of αvβ6 can reduce growth of xenografts [51,52,53] and can suppress experimental metastases [53]. Some one-third of carcinomas, the most common (85%) cancers, have elevated levels of αvβ6 compared to normal tissues [46,47,48,51,52,54]. Thus, therapeutic targeting of αvβ6 in carcinoma might be productive. In fact, as described below, our explorative biomarkers analyses have shown that colon carcinoma patients whose tumors expressed high-levels of αvβ6, when treated with the pan-αv blocking antibody abituzumab in combination with standard of care (SoC), may have a better response rate compared to patients with low-level αvβ6 expression [55]. By contrast, although most human pancreatic cancers express αvβ6 [56], when Hezel and colleagues treated transgenic mice that develop a model pancreatic adenocarcinoma (PDAC) with αvβ6-blocking antibodies, they reported that tumor progression was enhanced [57]. But since most clinical PDACs have deletion or dysfunction of SMAD4 [58], a transcription factor essential for the growth-inhibitory effects of TGFβ on epithelial cells, there is still a rationale for therapeutically targeting αvβ6 in PDAC.

What of therapeutic targeting β1 integrins expressed by solid cancers? Many β1 integrins have been pre-clinically implicated as promoting cancer-associated processes. Some β1 integrins have successfully been targeted pre-clinically, including αvβ1 [43], α2β1 [59], α5β1 [60], and α6β1 [61]. But currently only drugs specific for α5β1 have progressed to late stage trials for cancer therapy, excepting a pan-β1 inhibitory antibody P5, reportedly in phase 3 (PH3) trials for NSCLC. It seems that until a β1 integrin is identified that has sufficient selectivity for a process essential for the progression of a cancer, there are unlikely to be new β1 integrin-specific drugs developed. In the following section we provide an update on the current state of play of integrin targeted therapies especially in cancer.

## 2. Integrins Targeted in Clinical Trials

**Figure 1:**
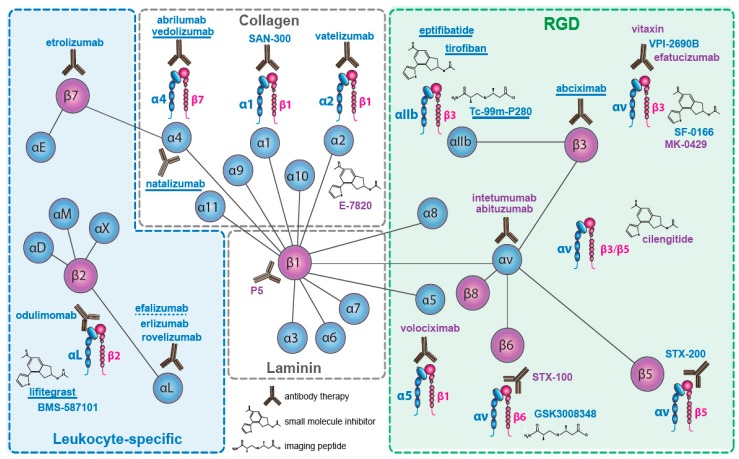
Integrins targeted in late stage clinical trials. The chains or heterodimers targeted, together with symbols indicating the type of drug used as mentioned in text are shown. Drugs named in violet have been used in cancer trials. Non-cancer drugs are named in blue. Marketed drugs are underlined; withdrawn drug indicated with broken underline.

Nineteen of the 24 integrin heterodimers, alone or as multi-chain families (e.g., αvβx; α4βx), have been therapeutic targets for drug discovery. Integrins which have been targeted are indicated in Figure 1. We will not consider the pharmacological strategies used, which have been detailed elsewhere (e.g., [62,63]), and we include only compounds that target integrins directly, rather than their ligands (e.g., excluding anti-fibronectin ED-B domain), or their downstream signaling pathways (e.g., anti-focal adhesion kinase). Similarly, we have omitted drugs whose integrin specificity emerges within a wide activity profile (e.g., AS-101 [64]). We have focused on late stage trials and on drugs that have reached the market.

We have identified some 480 drugs targeting integrins (the cut-off for our survey was July 2017). Where several companies have licensed a drug, target indication and stage of development may vary, with trials discontinued in one indication but still in progress in others. Here, we note the latest stage of clinical development, rather than stating that development has been discontinued. Since our last review [65], several late-stage trials have reported outcomes. The α4β7 inhibitor vedolizumab was launched for treatment of Crohn’s disease and ulcerative colitis (UC), and several other therapeutics targeting α4β7 have advanced into late stage trials. The αLβ2 inhibitor lifitegrast was launched as a treatment for dry-eye disease. Among less encouraging results, in cancer the PH3 and phase 2 (PH2) trials of the αvβ3/αvβ5 inhibitory peptide cilengitide in glioblastoma, in squamous cell carcinoma of the head and neck (SCCHN), and in non-small cell lung carcinoma (NSCLC), and of the αv-inhibitory antibodies abituzumab, in PH2 trials in colorectal carcinoma (CRC) and prostatic cancer, and of intetumumab in a melanoma and a prostatic carcinoma trial, failed their primary endpoints. Where late stage trial results have not been published, we generally cite the NIH Web-site (https://clinicaltrials.gov/) identifier locating the trial at ClinicalTrials.gov (NCTxxxxxxxx).

Integrins have also been targeted for diagnostic imaging. Apticitide (Tc-99m-P280), imaging gpIIbIIIa for acute deep-vein thrombosis in lower extremities, has been launched. (99m)Tc-maraciclatide, for identifying fibrosis in hypertrophic cardiomyopathy and acute coronary syndrome, and [18F]FPPRGD2 as a positron emission tomography (PET) imaging agent, for diagnosis of vascular inflammation diseases such as aortic aneurysm or carotid atherosclerosis, both imaging αvβ3, are in PH2 trials. 14 others imaging agents are reported to be in early development [66,67].

### 2.1. The Collagen Receptors

The four β1-series integrin collagen receptors (α1β1, α2β1, α10β1, α11β1) have each been therapeutically targeted, and for α2β1 there seems to be a clinical basis for further effort [68]. Only one program has targeted α10β1 and α11β1, but the development (by Xintela AB, Lund, Sweden) has apparently been abandoned.

• α1

α1β1 is a collagen-laminin receptor seen both as a target for inhibition of tumor angiogenesis and growth [69], and for the suppression of inflammation. The anti-α1β1 antibody SAN-300 (from Valeant (Quebec, QC, Canada)) is in PH2 trial for rheumatoid arthritis [70]. But active α1β1 can inhibit EGFR signaling [71]. Therapeutic inhibitors of EGF-receptor signaling are effective anti-cancer drugs (e.g., cetuximab; gefitinib; erlotinib). So therapeutic inhibition of α1β1 may hypothetically enhance EGFR signaling, and thus be pro-tumorigenic: it should be cautiously managed.

• α2

α2β1 is seen as an anti-thrombotic target [72], being responsible for activation of platelets and for their adhesion to damaged vessel walls [73,74]. It affects T-cell activation and survival [75] and regulates inflammation [76]. α2β1 is potentially also a target in cancer. It is implicated in promoting tumor cell invasion and proliferation [77]. Two α2β1 PH2 trials are active, and 8 clinical programs on α2β1 have been completed. Vatelizumab (CHR-1103), a humanized α2β1-blocking antibody developed by Chromos Molecular systems (Burnaby, BC, Canada) and now owned by Glenmark (Mumbai, India), was tested in a 96 week relapsing-remitting multiple sclerosis (MS) trial (ClinicalTrials.gov Identifier: NCT02222948). No clinical efficacy was reported, but there were no safety issues. Trials in inflammatory bowel disease (IBD) were also discontinued with no efficacy reported. E-7820 is a sulphonamide-based small molecule (developed by Eisai, Tokyo, Japan), whose action has been attributed to the reduction of α2 mRNA expression [78]. It showed little efficacy in several PH2 cancer trials targeting locally-advanced or metastatic colorectal carcinoma (CRC), combined with SoCs like FOLFIRI, , bevacizumab and irinotecan, or cetuximab [79].

The biology of α2β1 makes it an interesting therapeutic target, and as well-characterized inhibitors are known future clinical trials in oncology may be warranted.

### 2.2. The Fibronectin Receptors

Three β1 integrins (α4; α5; α8) and three αv integrins (β1; β3; β6) are receptors for fibronectin [1,80]. Five can bind at the Arg-Gly-Asp (RGD) sequence in the fibronectin type-III repeat 10, and one, α4β1, recognizes a Leu-Asp-Val-Pro—like (LDVP) sequence in the fibronectin type-III-CS1 region [81,82]. The role of four of these receptors in disease has been intensively studied (excluding αvβ1 and α8β1) [80]. An extensive preclinical literature links them to diverse human pathologies, including cancer, fibrosis, inflammation and autoimmune disease. Owing to the superficially well-defined ligand recognition sequences, they have become prominent targets for drug design. Yet it is not the ability to bind to fibronectin which has been their main feature of pharmacological interest.

• α4

α4β1 binds VCAM-1 in addition to fibronectin [1]. The α4 integrins (α4β1 and α4β7) regulate tissue invasion and homing of activated T-cells during inflammation. This is mediated both by V-CAM-1 (α4β1) and by MAd-CAM-1 (α4β7) [83,84]. Subsets of T-cells control inflammation in MS [85] and in IBDs, like Crohn’s [86], and UC [87], and blockade of α4-integrins can prevent tissue invasion of the activated T-cell populations driving these diseases [83]. Development of pan-α4 therapeutics was disrupted following clinical experience with natalizumab (Tysabri^®^) in therapy of MS. A fatal, virally-induced demyelination disease, progressive multifocal leukoencephalopathy (PML), rare in the immune-competent, arose in natalizumab-treated patients [88]. This challenged the safety of pan-α4-integrin blockade [89], and led to a largely successful quest for more specific inhibitors. Although low-molecular weight inhibitors of the α4 integrins can be made [90], only antibody-based therapeutics have so far reached the marketplace.

• Natalizumab

Natalizumab (Tysabri^®^; Antegren) is a humanized mouse monoclonal IgG4κ antibody recognizing the human α4 chain in both α4β1 and α4β7 [91,92]. It was developed by ELAN (Dublin, Ireland) and Biogen-Idec (Cambridge, MA, USA) after Yednock and colleagues showed that α4-blockade could inhibit experimentally induced autoimmune encephalomyelitis [93]. A pan α4-integrin inhibitor should in theory block the leukocyte diapedesis mediated by interaction of activated T-cells both with VCAM-1, on activated endothelia, and the homing mediated by MAd-CAM-1, the Peyer’s patch addressin, on the high-endothelial venules of the gut. X-ray structure of the α4β1-natalizumab-Fab complex revealed that the antibody did not alter α4β1 structure. It acted as an orthosteric inhibitor, binding adjacent to the VCAM-1 D1 domain-acceptor groove on the integrin. This may alter the orientation of the VCAM-1 D2 domain, so lowering the affinity of the VCAM-1-integrin interaction [94]. Such orthosteric inhibition resembles that predicted for αv-inhibition by abituzumab (see below).

Natalizumab in phase 1 (PH1) and PH2 trials had similar side effects to placebo, and in multi-center trials as a monotherapy for MS it strongly reduced the numbers of new lesions and relapses over placebo-treated patients [95,96]. It was registered for MS treatment as monotherapy or together with β-interferon in November 2004. However, it was withdrawn a year later when 3 treated patients developed PML under natalizumab therapy [95,97,98]. It was later recognized that this was due to an on-target side-effect. The blockade of T-cell patrolling and the consequent immune suppression in the brain led to induction of John Cunningham polyoma virus (JC-virus) and PML [88]. However, natalizumab was uniquely efficacious in MS and after extensive re-evaluation the US Food and Drug Administration (FDA) allowed its renewed use in MS under rigorous monitoring for JC-virus. An assay for anti-JC virus antibodies has allowed stratification of patients at risk. With these tools to hand, natalizumab is once again widely used for MS therapy, and that despite the severe consequences of treatment discontinuation [99]. It has also been tested in trials for rheumatoid arthritis, myeloma and Crohn’s disease, and in PH2 trials in cerebral ischemia, and stroke (ACTION trial) [100].

A Cochrane report concluded that there was benefit from natalizumab in Crohn’s disease trials, and it was successfully registered in this indication in January 2008 [101]. Biogen’s rapid responses to the severe adverse events of natalizumab are ethically commendable, and their subsequent collaboration with the FDA in response to patients’ wishes for the clinically effective but high-risk drug were also admirable [102]. Natalizumab is undergoing trials for myeloma and has also been approved for orphan drug use in relapsing-remitting MS.

• α5β1

Volociximab (EOS-200-4) is a humanized chimeric IgG4 antibody inhibiting the α5β1 integrin, and seen as an anti-angiogenic therapy for solid tumors and wet age-related macular degenerative disease. It was developed by PDL (Incline Village, NV, USA) and subsequently licensed to Biogen-Idec, Ophthotech (New York, NY, USA) and Abbott (Abbott Park, IL, USA), but development was stopped after lack of efficacy in PH2 trials [103,104]. Anti-angiogenesis therapy in cancer and ocular indications has been influenced by the wide successes of drugs targeting the VEGF pathway (e.g., bevacizumab) [105].

A pan- β1 murine monoclonal antibody, P5 (Chungbuk University (Cheongju, Korea)), claimed to predominantly act at α5β1, is reported in PH3 trials for NSCLC [106]. Development on the small molecule α5β1/pan-αv inhibitor GLPG-0187 (Galapagos (Mechelen, Belgium)) appears to have been stopped [107].

### 2.3. The αv—Integrins

αv integrins can control cancer biologies like tumor angiogenesis, growth, metastasis and immunomodulation [108]. They also influence the pathophysiology of bone metastasis, being expressed both on tumor cells and on osteoclasts [109]. The pattern of αv integrin expression varies between cancers. Malignant melanoma and glioblastoma cells, for instance, tend to express αvβ3, whereas in other indications like CRC and pancreatic carcinoma its expression is restricted to angiogenic endothelium [15,110]. By contrast, αvβ6 is expressed in many CRC tumors, but is absent from prostate carcinoma [55,111]. Furthermore, within an indication, both αv integrin expression level and subtype may vary within a patient population, and expression levels may vary within a given tumor [55].

Two pan-αv integrin antibodies have been evaluated in late-stage clinical trials, abituzumab (DI17E6, EMD 525797: Merck KGaA (Darmstadt, Germany)) [112] and intetumumab (CNTO95: Centocor (Malvern, PA, USA)) [113]. Both bind the αv subunit and inhibit all five αv integrins [114] (see Figure 1). The antibodies both inhibit ligand binding and tumor growth in xenograft models [112,115,116,117], but differ in their ability to trigger immune responses.

• Abituzumab

Abituzumab from Merck KGaA is a humanized, de-immunized monoclonal IgG2 antibody. It is deglycosylated, so it is expected to not induce antibody dependent cellular cytotoxicity. In PH1 trials in progressive castration-resistant prostate cancer (CRPC) patients with bone metastases after chemotherapy, it showed a favorable safety profile and some evidence of clinical activity [118]. In the subsequent PERSEUS PH2 trial on chemotherapy-naïve patients, with asymptomatic or mildly symptomatic metastatic CRPC with progressive bone lesions, patients were treated with abituzumab or placebo, in combination with luteinizing hormone-releasing hormone agonist/antagonist therapy. The primary endpoint, extended progression free survival (PFS), was not significantly different between the treatment groups. However, the cumulative incidence of bone lesion-progression was lower in abituzumab-treated patients [111].

ανβ5 and ανβ6 integrins are expressed in CRC cells [119,120], and over-expression of ανβ6 has been implicated as a negative prognostic marker associated with a reduction in median overall survival (mOS) in early-stage metastatic CRC (mCRC) [46,55]. Cross-talk between αv-integrins and TGFβ can regulate Epithelial-Mesenchymal Transition (EMT) [121]. αvβ6 locally activates TGFβ, to induce EMT and promote invasive and metastatic behavior of CRC cells [38,46,122].

In the POSEIDON PH1/2 trial, abituzumab was evaluated in mCRC [55]. The trial assessed the tolerability of abituzumab in combination with the SoC—cetuximab and irinotecan- at PH1, and explored the efficacy of the combination over the SoC (PH2) [55]. Abituzumab was acceptably tolerated in combination with cetuximab and irinotecan. The primary end point (PFS) was not met. A pre-planned biomarker analysis investigated the relationship between integrin expression and treatment outcome. Data suggested that high αvβ6 expression may be a negative prognostic marker for OS in the SoC cohort, and predictive for prolonged OS in the abituzumab combination cohort. Furthermore, the data indicated that the response to the abituzumab combination was improved in those patients with high integrin ανβ6-expression. Patients with low expression did not benefit from abituzumab. It is conceivable that abituzumab-based therapy might benefit mCRC patients with high αvβ6 expression on the primary tumor [55]. However, this must be verified in trials where levels of αvβ6-expression are used up-front for cohort-stratification.

Beside cancer, abituzumab is being investigated in a PH2 trial in patients with systemic-sclerosis-associated interstitial lung disease (SSc-ILD) (NCT02745145), to evaluate safety and efficacy (immune response). As in other fibrotic diseases, in SSc-associated fibrosis the data emphasize a role for TGFβ [123,124]. Local activation of TGFβ by αvβ6 might therefore alter disease progression in SSc-ILD. Such local and indirect inhibition of TGFβ by integrin inhibitors like abituzumab (pan- αv) or BG00011 (STX-100; αvβ6) might have a superior safety profile to systemic TGFβ inhibitors [124]. In addition, there may also be differential effects on disease progression produced by local inhibition of all αv integrins, compared with the inhibition of αvβ6 alone.

• Intetumumab

Intetumumab (CNTO-95) from Centocor is also a pan-αv integrin inhibitor. It is a fully human IgG1 and can induce ADCC, in contrast to abituzumab, an effector-negative IgG2. Several PH1 trials demonstrated a favorable safety profile for intetumumab. A PH2 trial in melanoma, as monotherapy and as combination with dacarbazine, demonstrated no statistically significant efficacy, but a trend for an improved OS [113]. Another PH2 trial investigated efficacy in combination with docetaxel and prednisone in mCRPC patients, but the intetumumab combination resulted in significantly reduced PFS over SoC at all end points, though without major effects on tolerability [125]. An antibody-drug conjugate, IMGN-388, is intetumumab bound to maytansinoid cytotoxic agent DM4, and has been investigated in a PH1 trial (NCT00721669).

• αvβ3

αvβ3 integrin has been preclinically implicated in tumor angiogenesis, macular degenerative eye disease, bone resorption, and in the progression of several tumors including melanoma and glioblastoma, where its expression is a negative prognostic indicator. Vitaxin (MEDI-523; AME (San Diego, CA, USA)), and efatucizumab (abegrin; MEDI-522: Medimmune (Gaithersburg, MD, USA)) are humanized higher affinity variants derived from murine antibody LM609. They have been tested in multiple PH2 oncology trials, for example in prostate (NCT00072930) and lung carcinomas, CRC (NCT00027729) and in melanoma (NCT00111696; NCT00066196), in combinations with standard of care [126]. But there has been no report of clinical efficacy, and their development appears to be quiescent.

Several other αvβ3 inhibitors are in PH2 trial in diverse non-oncological indications. SF-0166 (SciFluor Life Sciences (Cambridge, MA, USA)) is a fluorinated small molecule inhibitor in PH2 trial (NCT02914613) for topical treatment of wet age-related macular degeneration and diabetic macular edema. VPI-2690B (Vascular Pharmaceuticals (Chapel Hill, NC, USA) with Janssen (Beerse, Belgium)) is a monoclonal antibody in PH2 trial (NCT02251067) for diabetic nephropathy. MK-0429 (Merck and Co. (Kenilworth, NJ, USA)) is a small molecule in development both for hormone refractory prostate cancer and for osteoporosis [127], but no further development has been reported.

• αvβ5

αvβ5 is a vitronectin receptor which has been implicated in resorption of rod cell membranes [128], and as a co-receptor for adenovirus [129]. It is very widely distributed in normal human tissues [110,119]. Stromedix (Biogen-Idec) started development of a monoclonal antibody, STX-200, targeting sepsis, scleroderma and fibrosis, but no studies are reported in progress.

• αvβ3 and αvβ5

Cilengitide (EMD121974) from Merck KGaA is a constrained cyclic pentapeptide based on the RGD-sequence, that specifically inhibits αvβ3 and αvβ5 [130,131]. Preclinical studies supported its efficacy as an anti-angiogenic and anti-tumor agent—and it was active in αvβ3 and αvβ5-driven cellular assays at ~1 µM [108,131]. It suppressed breast metastasis to bone [132,133], and enhanced radiotherapy in orthotopic brain tumor models [134]. After unsuccessful early trials in pancreatic carcinoma and melanoma [135,136], a dose escalation PH1/2 trial in late-stage glioblastoma (GBM) showed some unexpected long-lasting responses as monotherapy [137] as well as an activity signal in combination with temozolomide in newly diagnosed GBM patients. This led to the first PH3 trial of an anti-integrin therapy in cancer, the CENTRIC trial (NCT00689221) in newly diagnosed GBM patients, in combination with radiation and chemotherapy. Patients were selected for their expression of the methylated- *O*^6^-methylguanine DNA methyltransferase (MGMT) gene promoter (a stratification marker for sensitivity to temozolimide), while a parallel PH2 study (CORE: NCT00813943) examined the temozolimide-insensitive MGMT-unmethylated population. Both trials showed that cilengitide had no clinical benefit. A PH2 trial (ADVANTAGE: NCT00705016) in first-line SCCHN patients, in combination with cetuximab and platinum-based chemotherapy also failed to give a clear positive outcome. A PH2 trial in first-line NSCLC (CERTO: NCT00842712), in combination with cetuximab and cisplatin, also failed its primary endpoint and development was stopped [138,139,140,141,142]. Trials altering the temozolimide regimen in GBM [143], with cediranib in recurrent GBM patients (NCT00979862) [144], in childhood progressive high-grade astrocytoma (NCT00679354) and in solid tumors with paclitaxel [145] were completed without significant clinical activity being observed. It may be noted that many other clinical PH3 programs using diverse approaches have failed for GBM over the past decade [146].

Cilengitide is rapidly cleared from serum (t_1/2_ = 3–5 h) [147], but continuous perfusion studies over 4 weeks with cilengitide at the limits of solubility (40 mg/h), attaining a steady state concentration of ~10 µM, also failed to improve the clinical outcome, without dose-limiting toxicity [148]. Preclinical studies suggested that nanomolar concentrations of cilengitide could on one hand activate αvβ3 and enhance angiogenesis and tumor growth [27], and on the other, enhance intratumoral vascular permeability and increase drug delivery [149]. This lead to much active debate about the role of αvβ3 and αvβ5 in tumor development (e.g., [150]). We should note here that the periodic cilengitide treatment in clinic, where nanomolar systemic concentrations occurred during therapy, did not adversely affect survival over controls in any trial.

The lack of efficacy of cilengitide in GBM trials was a surprise, as well as a disappointment [141], for there was no doubt of its preclinical efficacy as an αvβ3 and αvβ5 inhibitor and anti-angiogenic [131,151]. Furthermore, overexpression of αvβ3 in clinical GBM is a negative prognostic indicator [152,153]. However, the PH2 GBM trial conclusions were based on historical rather than blinded parallel controls. It is possible that the fast off-rate of cilengitide from its targets, rapid plasma clearance, or inadequate perfusion of the brain tumor environment suppressed any potential clinical efficacy in the CENTRIC trial. A continuous infusion study which supplied high doses of the drug (stable systemic levels ~10 µM) was not performed in the context of radio-combination therapy in GBM [148]. So in future, if the correct clinical context can be discovered, cilengitide, which had a rather innocuous side-effect profile, might yet become an effective therapy.

• αvβ6

αvβ6, originally identified on airway epithelial cells, and as a fibronectin receptor on colon carcinoma cells, was shown in a landmark study to be a local activator of latent TGFβ, and lack of αvβ6 in mice startlingly phenocopied the TGFβ1 knock-out mouse [38] (see Introduction). In mCRC patients treated with abituzumab, a pan- αv inhibitor (see below), an extended OS appeared to be correlated with high tumor expression of αvβ6, independent of αvβ3, αvβ5, or αvβ8 expression [55]—implicating inhibition of αvβ6 as the causal target.

Several companies are developing antibody and small molecule inhibitors of αvβ6. The humanized monoclonal antibody STX-100 (BG00011) from Biogen-Idec [52,154] is in PH2 trials for idiopathic pulmonary fibrosis and for nephropathy.The trial (NCT01371305) has completed (May 2017) but no results have been reported to date. GSK3008348 (GSK (Brentford, UK)) is an αvβ6 PET-imaging agents in PH1 trial for therapy of idiopathic pulmonary fibrosis (NCT03069989). An antibody-drug conjugate targeting αvβ6 is in early development (15H3: Seattle Genetics (Bothell, WA, USA)) but clinical trial results have not been reported.

• αvβ1 and αvβ8

αvβ8 is a LTGFβ activator, but its tissue distribution differs markedly from αvβ6 [155,156]. Efforts to specifically target αvβ8 have not passed the preclinical stage—however, it is also potentially a target for anti-fibrotic therapies. αvβ1 has remained a mysterious integrin because as yet only biochemical methods, rather than antibodies, can specifically identify its distribution in tissues. Nevertheless, recent studies suggest that it too can activate LTGFβ and play a critical role in tissue fibrosis [43,45]. It may be an interesting target for therapy.

### 2.4. The Laminin Receptors

Three β1 integrins (α3; α6; α7), and α6β4 are laminin receptors; two collagen receptors (α1; α2) (see Section 2.1.) may as a minor function also bind laminins [1,80]. Laminins are major components of basement membranes. Their primary integrin receptors support cell homeostasis in the adult epithelia and endothelia, tissue development during embryogenesis, and the altered interactions that accompany pathological changes, including during cancers. The 16 laminin isoforms, combine with the multiple extracellular and intracellular splice variants of α3, α6, and α7 that modify their binding specificities and signalling [157]. The precise binding interactions with integrins are still not completely defined [158,159]. However, it appears from preclinical models that these integrins may both promote and inhibit tumor development, depending on tumor type and stage [157]. At present no drug development project specifically targeting the laminin receptors has reached clinical trials, ignoring the pan-specific anti-β1 antibody, P5 [106].

### 2.5. The Leukocyte Integrins

#### 2.5.1. β2 Integrins

Over the past decade many structural details of the conformational activation cycles that integrins experience have been revealed. Here, cytoplasmic signals trigger the transformation of integrin structures which cannot bind extracellular ligands, to high-affinity states that can (“inside-out” signaling) [160,161,162,163]. Ligand binding to the high-affinity forms then induces intracellular (“outside-in”) signaling [4]. Drug developers must now confront the question of which of these states to target and how to target them. This issue is exemplified by the β2 and β3 series integrins.

β2-series integrins are restricted to leukocyte lineages, where they regulate cell-cell interactions involved in innate and adaptive immunity. The low-affinity state on leukocytes in the circulation is rapidly converted to a 10^4^-fold higher affinity in response to local cytokine signals [164,165]. β2-series integrins mediate trapping and rolling of leukocytes at the endothelial surface [166]. Other than their role in homeostasis, β2 integrins control a wide range of human pathologies, and their dysfunction drives many autoimmune and inflammatory diseases (reviewed in [162]). Inhibitory antibodies and small molecule β2-integrin inhibitors have been generated, but enthusiasm for them as systemic targets has become muted of late, coinciding with the withdrawal of efalizumab with those PML issues previously observed in natalizumab trials [167].

• Lifitegrast

Lifitegrast (Xiidra^®^; SAR 1118) is a low-molecular weight tetrahydroisoquinoline antagonist of αLβ2 [168], originally from Sunesis Pharmaceuticals (San Francisco, CA, USA) [169]. It was launched in August 2016 as a treatment for xerophthalmia (dry-eye disease), following out-licensing to Shire Pharmaceuticals (Lexington, MA, USA) and the OPUS2 PH3 trial (NCT10743729) [170]. Lifitegrast blocks T-cell invasion of the conjunctiva, and suppresses local inflammation. Lifitegrast applied as a 5% eye-drop solution is rapidly adsorbed into ocular tissue, and systemic clearance is rapid (plasma t_1/2_ = 47 min in rat; <4 h in human). This, and its low potency in CYP450 inhibition assays and *ether-a-go-go* studies, predicted the observed good drug tolerance in humans (SONATA trial: NCT01636206)—without the side-effects of systemic inhibitors of B- and T-cell adhesion [88,171].

• Efalizumab

Efalizumab (Raptiva^®^) is a humanized anti LFA-1 (αLβ2) antibody targeting the αL (CD11a) chain of the complex. Discovered by Genentech, it was developed and launched by Serono (Geneva, Switzerland) in November 2003 for treatment of plaque psoriasis, where it was an effective therapy [172]. However, the drug was withdrawn in 2009 after treatment of over 45,000 patients, when it was associated with 3 cases of PML [171]. Unlike natalizumab in MS (see above), alternative psoriasis therapies were available, and the risks of using Efalizumab were considered to outweigh the benefits.

• Odulimomab

Odulimomab is a chimeric IgG1 antibody developed by Genzyme (Cambridge, MA, USA) for targeting LFA-1 to prevent delayed graft function. It reached PH3 trial in the clinic. Although preclinical studies support the efficacy of anti-LFA-1 antibodies in suppressing graft rejection [173,174], Odulimomab development appears to have been terminated.

• Rovelizumab

Rovelizumab (Leukarrest^®^) is a humanized anti-αL antibody (Hu23F2G) developed by ICOS (Bothell, WA, USA) which entered PH3 trial for cerebral ischemia. Development appears to have been stopped [175]. Given its anti-macrophage function, it has been suggested that post-irradiated tumors might be a better therapeutic target for such a drug [176].

• Other β2 drugs stopped in PH2

Genentech designed erlizumab, a humanized anti-αL antibody, as a therapy for hemorrhagic shock, in collaboration with Roche (Basel, Switzerland). It failed its PH2 trial endpoints, and development was stopped. BMS-587101, a small molecular weight inhibitor of αLβ2 designed for topical use in psoriasis, was stopped after showing signs of hepatotoxicity [177].

Inhibitors of β2 integrins work by specifically inhibiting cell adhesion and trapping of white blood cell lineages, mechanisms distinct from those of other immunosuppressive drugs: thus they are potentially attractive therapeutics, as summarized above. However, obviously therapeutic strategies must avoid life-threatening systemic side-effects. The success of Lifitegrast indicates one possible approach: topical application combined with short systemic half-life to limit exposure to the drug. Where this is not possible, focusing the therapeutic effect to a precisely defined activation state of the target integrins may also prove a successful way of minimizing undesired toxicities in future, and to enable the design of inhibitors which do not induce pro-adhesive alterations [63,178,179]. For example, it is possible to stabilize the chelation of anti-adhesive Ca^2+^ ions at the β2 MIDAS site [180], and it may be possible by suitable drug design to lock the β2 integrins in a closed low affinity conformation.

#### 2.5.2. β7 Integrins

• Vedolizumab

Vedolizumab (Entyvio^®^) is a humanized antibody IgG inhibitor of integrin α4β7, originally developed by Millenium pharmaceuticals (Cambridge, MA, USA) as MLN002, and launched in May 2014 after successful PH3 trials in patients with TNF-resistant Crohn’s disease and UC [181,182]. Further late-stage clinical trials are in progress, including for steroid-refractory acute intestinal graft vs. host disease, in fistulizing Crohn’s disease and in chronic pouchitis.

Vedolizumab specifically inhibits the interaction of α4β7 with MAd-CAM1. This blocks the homing of cytokine-activated T- cells to the high-endothelial venules of Peyer’s patches in gut [183]. No induction of PML has been reported for vedolizumab [182], which unlike natalizumab does not block T-cell patrolling in brain. This enhances the risk-benefit profile for vedolizumab, and may extend its future utility in inflammatory gut diseases.

αEβ7 integrin (CD103-β7) is concentrated on dendritic cells (DCs) in the intestine, and on intraepithelial T-cells, where it mediates T-cell adhesion and DC directed retention in the gut [183]. Etrolizumab is a recombinant humanized IgG1 monoclonal antibody developed by Genentech (San Francisco, CA, USA), specifically binding to the β7 chain and blocking α4β7 and αEβ7 integrins, an inhibition profile that distinguishes it from natalizumab and vedolizumab. It is in 8 PH3 trials in similar indications to vedolizumab (e.g., UC: NCT02118584; Crohn’s: NCT02118584), and its specificity may reduce side-effects compared to natalizumab and vedolizumab [87].

Abrilumab (AMG-181: MEDI-7183) is a human IgG inhibitor of integrin α4β7, developed by Amgen (Thousand Oaks, CA, USA) with AstraZeneca (Cambridge, UK), for treating UC and Crohn’s disease, and considered for CRC [184]. Development was discontinued in 2016 for “strategic reasons”, following PH2 results which did not show the necessary clinical profile.

A meta-analysis of opportunistic infection and malignancy, which are a concern where immune suppressive strategies are used, in 12 Crohn’s disease and UC clinical trials using α4 and β7 inhibitors found no significant enhanced risk [185].

### 2.6. β3—Inhibition on Platelets

The integrins gpIIbIIIa (αIIbβ3) and αvβ3 have been therapeutic targets. GpIIbIIIa for its role on platelets during thrombosis [11,186], and αvβ3 for its proposed roles in tumor angiogenesis and proliferation [108]. Both have been clinically targeted by antibody-, peptide- and small non-peptidic-drugs with variable therapeutic success. Both integrins bind protein ligands at RGD-peptides, and drug development programs focused on tuning inhibitor selectivity by modifying distance, stereochemistry and steric constraints between acidic and basic centers, mimicking Asp and Arg respectively. The X-ray structures of αvβ3 and of gpIIbIIIa solved in the presence and absence of specific inhibitors helped clarify the empirical data set [187,188,189,190]. In drugs targeting gpIIbIIIa, the basic center must stretch further into the β3 chain than in αvβ3, in order to coordinate the MIDAS metal ions in the beta-chain A-domain (“I-domain”) , and the substitution of a hydrophobic residue in gpIIbIIIa (Phe231) for a charged one in αvβ3 (Asp218) permits the binding of more hydrophobic aliphatic chains [188,190].

• GpIIbIIIa Inhibitors

Anti-thrombotics fall into three major classes: fibrinolytics, anti-coagulants, and platelet aggregation inhibitors (PAIs), targeting surface proteins [191]. GpIIbIIIa on resting platelets has low affinity for fibrinogen. It is rapidly converted to a high-affinity form following platelet activation by thrombin, collagen or ADP. This high affinity form induces platelet aggregation during blood clotting and in pathological thrombosis, as revealed by the gpIIbIIIa-deficiency disease Glanzmann’s thrombocytopenia [11]. GpIIbIIIa antagonists are more effective PAIs in vitro than either aspirin or ticlopidine alone [192]. And the mechanism of action of these inhibitors, respectively by cyclo-oxygenase inhibition and by ADP-receptor antagonism is distinct from the gpIIbIIIa antagonists.

As noted earlier, integrins function bidirectionally, being activated inside-out by intracellular signaling, and by outside-in by extracellular stimuli [193]. Integrin antagonists that act at the ligand binding site may counter-intuitively agonize outside-in signaling from the receptor, although they antagonize receptor binding to extracellular ligands [190,194,195,196].

Despite concerns that competitive gpIIbIIIa antagonists might induce thrombosis or enhance bleeding, this has not been a major issue for gpIIbIIIa inhibitors in the clinic [197]. Bleeding events with gpIIbIIIa inhibitors are related rather to acute thrombocytopenia, induced by antibodies against drug-target complexes [198]. The historical trials investigating these issues and a detailed overview of the pharmacology of gpIIbIIIa inhibitors is excellently reviewed elsewhere [199].

• Abciximab (c7E3Fab; ReoPro)

The pan-β3 inhibitory humanized antibody Fab fragment, Reopro^®^, was the first integrin inhibitor to reach the therapeutic market. It was registered by Centocor and Eli Lilly (Indianapolis, IN, USA) in January 1995 for prevention of clot formation during high-risk coronary angioplasty. The murine progenitor acts allosterically at the gpIIIa chain (integrin β3 subunit) [195,200]. The continued success of ReoPro as an anti-thrombotic is indicated by the biosimilars (Rexipro; Clotinab; Abcixirel) that have since been launched. Although ReoPro targets the β3 chain and inhibits both gpIIbIIIa and αvβ3 no specific effects have been attributed to its action on αvβ3. Platelets halt bleeding in wounds as well as pathologically triggering thrombosis, so gpIIbIIIa inhibitors must act in a narrow therapeutic window to prevent uncontrolled bleeding. Indeed, a primary adverse effect seen in ReoPro therapy is thrombocytopenia [201]. However, its benefits in anti-thrombotic therapy are clear, and extended bleeding times are often considered clinically acceptable.

Abciximab was originally launched as a surgical-adjunct therapy for anti-thrombotic prophylaxis during percutaneous coronary intervention (PCI) (high-risk coronary angioplasty). As it was well tolerated in the clinic, its use was extended to all angioplasty, and to unstable angina patients due for angioplasty. It was registered for use in unstable angina, and subsequently a biosimilar was also launched for acute myocardial infarction by ISU-ABXIS (Seongnam-si, Korea) under the name Clotinab^®^ [202].

Following the clinical success of Abciximab many companies attempted to develop low-molecular weight orally-available inhibitors of gpIIbIIIa. This seemed straightforward given the existing knowledge of integrin biology. However, orally available drugs that inhibited activated gpIIbIIIa receptors often increased bleeding events, leading to severe thrombocytopenia [198,203]. Nevertheless two low molecular weight parenteral gpIIbIIIa inhibitors were successfully developed, Tirofiban and Eptifibatide.

• Tirofiban (Aggrastat^®^)

Tirofiban (*N*-(butylsulfonyl)-*O*-(4-(4-piperidinyl)butyl)-*L*-tyrosine mono hydrochloride monohydrate) was launched by Merck and Co. in August 1998 for use in acute coronary syndrome. It was subsequently registered for prevention of myocardial infarction in unstable angina or non-Q-wave myocardial infarction. It has recently been approved for use in percutaneous coronary intervention, and has been sold under license by a number of companies. It has a short plasma half-life of 2 h [204,205].

• Eptifibatide (Integrilin^®^)

Eptifibatide is a cyclic hexapeptide peptide inhibitor developed by COR therapeutics (Millenium/Takeda (Deerfield, IL, USA)) which is an active-site mimetic of the snake venom disintegrin Barbourin [167,206]. In Eptifibatide, homo-Arg substitutes for Lys in the Barbourin KGD- sequence. Eptifibatide was launched in July 1998, and used to treat unstable angina, acute coronary syndrome and myocardial infarction [207].

The structural basis for Tirofiban and Eptifibatide inhibition was elegantly revealed using X-ray crystallography [190]. Although the molecules act in a similar manner, they can show differential biological activities [208]. Whereas gpIIbIIIa inhibitors block interaction of platelets with fibrinogen, they do not prevent platelet activation, as does Aspirin, and their effects can rapidly wear off unless, like ReoPro they show a depot effect [202].

Tc-99m-P280 (Apcitide^®^) is a gpIIbIIIa-binding peptide launched in 1998 for the imaging of deep vein thromboses. It is the only approved reagent for this indication. It is supplied as a kit for use with a Technicium-99 generator, so its usage may be constrained by current difficulties in the supply of molybdenum-99 [209].

• Pharmacology of gpIIbIIIa inhibitors

Reopro^®^ is a Fab fragment with a short plasma half-life (~4 h) and a low volume of distribution. It has a high affinity for its target (Kd~5 nM), and is rapidly sequestered by circulating platelets (~50% bound within 10 min). Yet 13–29% of available gpIIbIIIa is still blocked by Reopro^®^ a week following infusion, likely due to the depot-effect from the gpIIbIIIa pool on platelets, which hinders more rapid systemic clearance [199,210]. The low-molecular weight inhibitors, by contrast, have fast-off rates and are rapidly cleared, both systemically and from the target.

After the enthusiastic reception of Reopro^®^, and with an accessible chemistry, a burst of low-molecular weight inhibitors of gpIIbIIIa were discovered, both orally available and parenteral, but development of these (66 programs) has halted. The oral inhibitors often agonized gpIIbIIIa [202].

The future for gpIIbIIIa inhibitors is uncertain, due the use of effective anti-thrombotics from other drug classes. Factor Xa-and thrombin- inhibitors are competing in the market for gpIIbIIIa inhibitors [191]. Orally available pure antagonists of gpIIbIIIa with a better tunable therapeutic window might in future alter this situation, but meanwhile gpIIbIIIa inhibitors are niche drugs.

## 3. Discussion

Integrins control complex biologies that modulate cell-cell and cell-extracellular matrix interactions, and also the activation of some TGFβ family members. Integrins are exposed at the cell surface, and accessible to many forms of therapeutic intervention. Furthermore, their expression is regulated, often dramatically, during human malignancies, and during fibrotic and inflammatory diseases. This has made them seductive therapeutic targets. Yet nearly 30 years of interaction has cooled the ardor of therapeutic developers for many of these previously desirable partners.

Both our knowledge and our ignorance of integrin biology have grown with time. There are several reasons for this. First, surprisingly, we still lack a reliable quantitative normal and pathological tissue distribution for many integrins. We have been restricted by a lack of antibodies which reliably function in immunohistochemistry (IHC) of formalin-fixed-paraffin embedded (FFPE) material, the material that makes up the majority of human pathology collections. The collection, storage and archiving of frozen tissues, where many antibodies do reliably function, is challenging, not least in the context of a multi-center, multi-continent clinical trial. We have been restricted by the persistence of archaic analytical modalities, rather than quantitative digital image analysis for enumerating relative expression levels in situ. Integrin RNA expression-profiling is a poor surrogate, as integrins are heterodimers. Furthermore, antibodies capable of detecting integrins in FFPE material rarely reveal the activation state of integrins in situ. Yet this defines whether they are drug targets helping drive a pathology (e.g., gpIIbIIIa on activated platelets), or whether they are by-standers sustaining homeostasis (e.g., gpIIbIIIa on circulating platelets). The availability of antibodies that can recognize different activation states of gpIIbIIIa have greatly helped interpret therapeutic responses [211]. Second, we have been restricted by the complexities of integrin biology, especially regarding the diverse structural configurations, reflecting different activated and signaling states—and this has restricted our knowledge of their particular relevance in a given disease. Finally, the complexities of integrin biology continue to confound the naïve observer: from their startling role in LTGFβ activation [212] to their emerging functions on exosomes as enhancers of the metastatic niche [213].

What has started to emerge from the nearly 70,000 papers referring to integrins in MEDLINE, is a theme replaying a current trend in cancer biology. Reagents that have effectively targeted biological mechanisms driving in vitro and in vivo models have not translated to efficacious therapeutics in the clinic—though it must be remembered that this statement is based largely on combination therapies with chemotherapeutics, rather than on modern immunotherapies. This includes biologies like angiogenesis, targeting endothelial cells or podocytes (blocking of αvβ3, αvβ5, or α5β1), or cell proliferation, targeting tumor cells or fibroblast lineages (blocking αvβ3, α5β1, α9β1, or α2β1). Whereas drugs that have targeted immune-cell-cell interaction targeting T-cells or B-cells (α4β1, α4β7, αLβ2, αxβ2) have clear clinical efficacy. In total, the statistically valid numbers of clinical trials on which this comment is based is still quite low, and this might be a chance observation. Clarification will await further studies. Predictive biomarker for patient stratification could also be a major step forward for the integrin field to translate preclinical efficacy into a beneficial treatment for patients.

Despite substantial effort, only seven drugs targeting integrins have actually reached the clinical market: abciximab; tirofiban; eptifibatibe; natalizumab; vedolizumab; and lifitegrast; with efalizumab being withdrawn. This seems somewhat below par for drug development, as 10% of drugs entering PH1 trails are reckoned on average to successfully pass PH3 into the market. As mentioned, some 480 drugs have entered integrin clinical trials, and some have been tested in multiple indications. Why this apparent lack of success? On one hand, animal models and especially systemic knock-out mouse models have been misleading for the integrins—excepting, perhaps, the models where overt immune function was involved. Embryonal function of an integrin need not reflect its function either in the normal or the ill adult. For example, the αv-integrin constitutive knockout mouse was 100% embryonal-perinatal lethal [214]: meanwhile humans subjected to systemic pan-αv inhibition with the antibody abituzumab remain healthy over years—within the context of their diseases—and without significant increased drug-induced mortality [55].

Prospective cancer therapies have often been investigated using xenograft models. The limited translatability of such cell-line derived tumor models to human disease has been extensively discussed, and is one of the many contributory factors to the lack of reproducibility of preclinical cancer studies [215]. The selection pressure imposed during the establishment of a cancer cell line, and from it an in vivo model, moves it far, far away from the situation in patients [216,217]. Orthotopic or patient-derived xenograft explant models produce more translatable preclinical data, and so may be more informative here [218].

The pharmaceutical industry appears conservative. This is understandable as getting a cancer medication from concept to market typically costs over a billion euros, dollars or pounds. This has left a number of interesting technologies relatively untested in the field of integrin therapeutics. For example antibody-drug conjugates, small-molecule-drug conjugates, co-therapy with immune-modulators, and more exotic antibody technologies, using smaller and so more tissue permeable single chain antibodies. Intrabodies, bi- or multi-paratopic antibodies to alter multiple functions simultaneously, or the use of alternative-binder technologies, like the knottins, DARPins and affimers [219] have yet to be therapeutically explored in the integrin field. At present, affinity-reagent targeted drug delivery is enjoying a renaissance, and has not been extensively exploited clinically in the integrin therapeutic area. Several integrins may be interesting targets for such an approach. As ever, it remains to be seen whether our knowledge of integrin distribution, our ability to target precisely enough, and the internalization and intracellular trafficking response of the targeted integrins in particular pathologies will allow effective and safe therapies to be generated.

• Health Economics: Will New Integrin-Targeting Drugs Be Affordable?

As we have described here, there have been several therapeutic anti-integrin successes. As antibody-based therapeutics are seen as transforming treatment success rates, especially in cancer [220], the possibility that integrin targeting might increase therapeutic success should be applauded. However, while antibodies can achieve marked clinical benefits (e.g., anti-checkpoint inhibitors [220]) the annual drug costs of treating an oncology patient can easily exceed $100,000 in the USA [221]. Generating an FDA-approved drug [222,223] costs $1.5–2.5 billion, including the costs of the >90% attrition of other drugs in company pipelines [222]. Regardless of this, the final cost to the user is determined by what the market will bear [224]. Therefore, anti-integrin antibody approvals by the FDA may be priced as leading oncology drugs, since these are established tolerated-costs for life threatening diseases.

Consider this: NCI cancer-incidence figures [225] estimate over 1.1 million new cases of the common carcinomas for 2017 in the USA, so, for example, considering a drug targeting αvβ6 integrin, which is expressed by about one-third of all carcinomas [54]. If 30–40% of these patients (high αvβ6 expressers [46,48,51]) benefited from αvβ6-bocking therapy, the potential annual cost at $100,000 each, to the state would be $11–14 billion, for a single drug, in the USA alone.

Does this make development of anti-integrin therapies pointless? Hopefully not. The spiraling cost of drugs (increasing 10% per year till 2013 [223]) is forcing national customers to establish cost-benefit guidelines addressing pharmaceutical companies. For example, in the United Kingdom (UK) the National Institute for Health and Care Excellence (NICE) determines which drugs and technologies may be used by the National Health System. As with most health-economic groups they measure clinical effectiveness in Quality-Adjusted Life Years (QALY) where one QALY is one year of healthy life. An approved drug usually costs no more than £20–30,000 per QALY, although end-of-life treatments that add 3–24 months of life can cost up to £50,000 annually. To meet these guidelines NICE negotiates significant discounts directly with pharmaceutical companies. By contrast, the USA health service, the Center for Medicaid and Medicare Services, does not have the same freedom to negotiate similar discounts. So in the USA drugs tend to cost more than in UK—and more than anywhere else in the world. For example, Regorafenib (a kinase inhibitor) treatment of mCRC costs $900,000 per QALY [226]. A regrettable consequence is that if pharmaceutical companies base negotiations on USA pricing, effective drugs may become inaccessibly costly both for national health systems, and potentially also for patients.

This is undoubtedly neither a mutually desirable, nor an acceptable, nor even a necessary outcome.

Thus, even though the development costs of drugs is still expected to rise for the foreseeable future, the increased availability and approval of cheaper generic drugs (for small molecules) and biosimilars (for biotherapeutics) (discussed in [226]) and especially any future revision of USA policy regarding negotiation with pharmaceutical companies, may result in far wider access to effective therapeutics, to the great benefit of patients.

## 4. Conclusions

Integrins have been a focus of therapeutic development for nearly 30 years, but despite some outstanding therapeutic successes, their complex biology has often confounded drug development. This is especially true in cancer therapy. While “simple” cell-cell interactions have been effectively targeted, a drug designed to target other specific biological mechanism may be useless when faced with less obvious functions driven by an integrin target. For example, the discovery of exosomes as controlling elements in cancer occurred after most current integrin cancer trials were initiated. Their significance and what the consequences of inhibiting their function might be, or might have been in past trials, remains a blank page. Now that knowledge of integrin structure can enable the design of drugs specific to given integrin activation states, we may see more successful future developments, including in cancer therapy targeting integrins.
